# Application effect of extensively hydrolyzed milk protein formula and follow-up in preterm children with a gestational age of less than 34 weeks: study protocol for a randomized controlled trial

**DOI:** 10.1186/s13063-015-1030-5

**Published:** 2015-11-04

**Authors:** Li-Ping Yin, Li-Juan Qian, Huan Zhu, Yan Chen, Han Li, Ji-Nan Han, Li-Xing Qiao

**Affiliations:** Department of Paediatrics, Zhongda Hospital Southeast University, 87 Dingjiaqiao, Nanjing, 210009 China

**Keywords:** Extensively hydrolyzed formula, Preterm children, Feeding intolerance, Growth and development, Follow-up

## Abstract

**Background:**

The average incidence of preterm birth in the world is up to 11.1 %, and deaths of preterm children account for more than 50 % of neonatal deaths. Gastrointestinal function of preterm children with a gestational age less than 34 weeks is immaturely developed. For preterm children who can only be fed with formula due to their mothers’ sickness, choosing a suitable formula can not only meet the high nutritional needs of preterm children, but also solve their low gastrointestinal tolerability, and is thus very important.

**Methods/Design:**

The study is a prospective, randomized, single-blind and controlled clinical trial. Preterm children with a gestational age less than 34 weeks meeting the inclusion criteria who cannot be breastfed will be included. To demonstrate the application effect of extensively hydrolyzed milk protein formula on the target population, preterm children will be randomized into two groups, 185 subjects in each group. The observation group will be fed with extensively hydrolyzed milk protein (100 % whey protein) formula, while the control group will be fed with preterm children’s formula until the children are discharged from the neonatal intensive care unit (NICU). All the formula involved in this study will be from Dumex. After discharge, both groups will be uniformly fed with formula for 0 to 6-month-old infants. For statistical analysis, a chi-square test and Student’s *t* test will be applied using SAS 9.4.

**Discussion:**

This will be the first randomized controlled clinical study with long-term observation of the growth and development of preterm children during the NICU stay and at 3-month follow-up after discharge from the NICU. Results from this study will be used to determine whether the extensively hydrolyzed formula is more suitable for the low gastrointestinal tolerability of preterm children, and also whether feeding preterm children who are fed with such formula during the NICU stay with ordinary infant formula after discharge from the NICU would affect the normal growth and development of preterm children in the early stage of their lives.

**Trial registration:**

This study was registered with the Chinese Clinical Trial Registry (http://www.chictr.org.cn/) with number ChiCTR-IOR-14005696, on December 22, 2014.

## Background

The neonatal period is a special period of life, in which the infant gradually transforms from complete dependence on the mother for survival into a mature individual. In 1976, the World Health Organization (WHO) defined preterm children as newborns with a gestational age of <37 weeks (<259 days) [1]. In 2005, the American Academy of Pediatrics and the American College of Obstetricians and Gynecologists [2] defined newborns with a gestational age of 34–36^+6^ weeks as late preterm birth, and less than 34 weeks as early preterm birth; in 2012, the WHO [3] defined preterm children with a gestational age of <28 weeks as extremely preterm birth, 28–31^+6^ weeks as very preterm birth, 32–36^+6^ weeks as moderate preterm birth, and 34–36^+6^ weeks as late preterm birth. The incidence of preterm birth varies greatly in different countries. In 2010, the incidence of preterm birth in 11 countries worldwide was ≥15 % [4]. The average incidence of preterm birth all over the world is up to 11.1 %. A total of 14.9 million preterm children are born worldwide every year [5], and in these preterm live births, more than 1 million preterm children eventually die [6], accounting for more than 50 % of neonatal deaths. In China, there is little data on the incidence of preterm birth nationwide. The WHO reported that the incidence of preterm birth in China in 2010 was <10 % [4]. The number of preterm children in ten countries including China accounts for 60 % of the world’s total. The mortality of preterm children in China is very high. From 2000 to 2008, preterm children led to 14–15 % of deaths of children younger than 5 years old in China [7]. The younger the gestational age, the lower the birth weight is and the higher the mortality is. According to the above data, we find that in all medical problems of newborns, the improvement of the survival rate and quality of life of preterm children is the target that the world focuses on tackling.

**Fig. 1:**
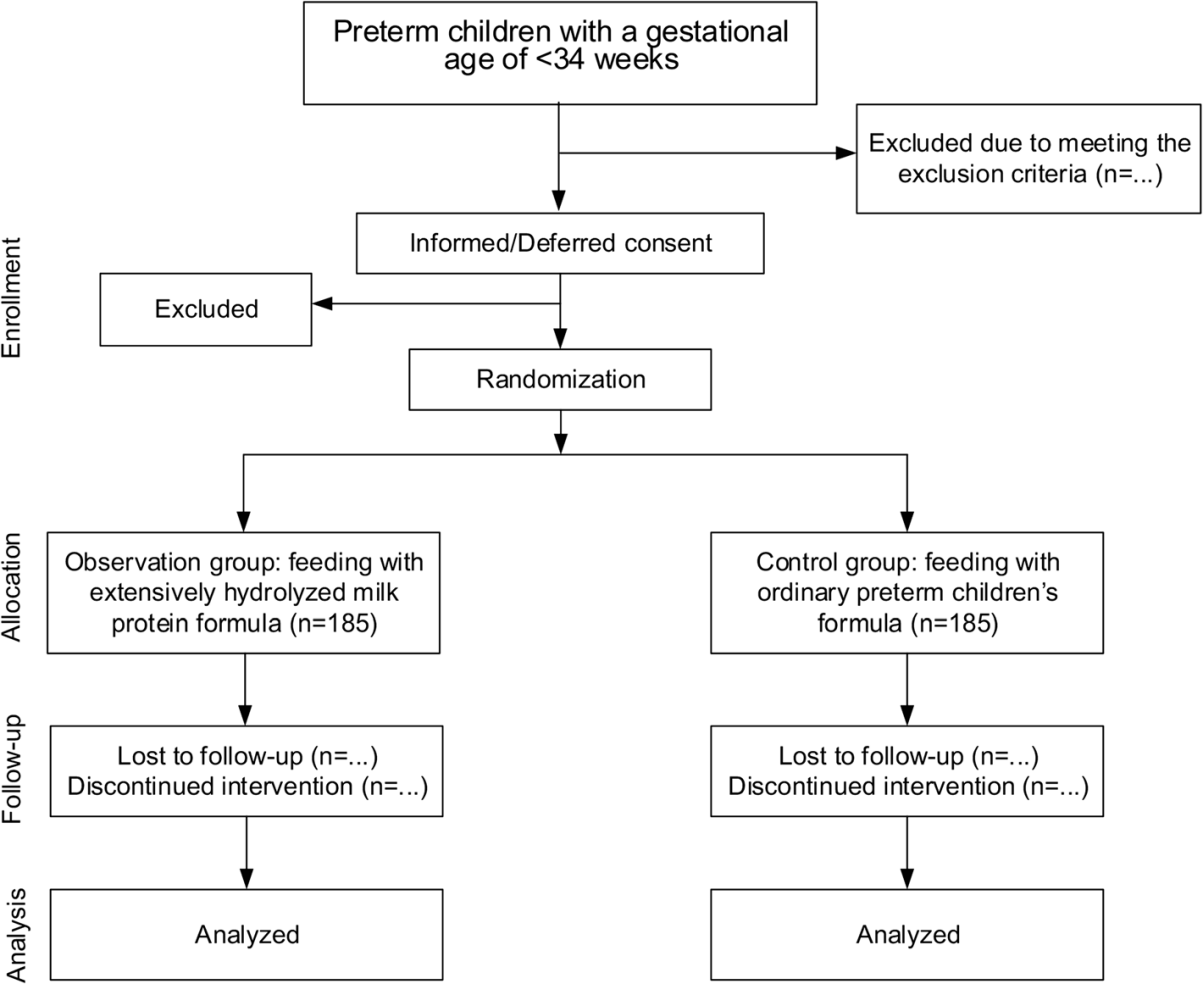
CONSORT flowchart of subject enrollment

The fetus in utero obtains nutrition through the placenta as a parenteral nutrition (PN) pathway. After birth, the newborns need to switch to enteral nutrition, while the completion of enteral nutrition requires effective and coordinated sucking-swallowing, gastric emptying, intestinal peristalsis and the digestion and absorption of nutrients. However, these functions of preterm children are immature, and the younger the gestational age, the more immature these functions. First, the increase in the extensibility and absorption area (microvilli) of the gastrointestinal tract of preterm children is mainly completed in the last 3 months of pregnancy [8]. Second, the sucking-swallowing-breathing action of preterm children with a gestational age of less than 32 weeks is much more uncoordinated; only children with a gestational age of 34–35 weeks have coordinated nutritive sucking [9]. In addition, the gastrointestinal motility of preterm children is weak, and the duodenum of preterm children with a gestational age of <32 weeks lacks the spread of migrating motor complex (MMC) [10]. Until after the gestational age of 34 weeks, clear and ever-increasing MMC gradually emerge. The above mechanisms lead to prolonged gastric emptying time and intestinal transit time of food in preterm children, and thus preterm children with a gestational age of less than 34 weeks are prone to gastroesophageal reflux, feeding intolerance (FI), delayed meconium drainage, intestinal dilatation and other phenomena. In addition, the development of digestive enzymes in preterm children is also very immature, and preterm children have inadequate secretion and low activity of gastric acid, gastric protease-pepsin and pancreatic protease, which weaken the ability of the gastrointestinal tract to hydrolyze protein into peptides and amino acids [10]; the concentration of pancreatic lipases and duodenal bile acids is very low [11], which reduces the intestinal absorption of lipids, especially the long-chain fatty acids (LCT). Therefore, preterm children are also prone to extrauterine growth retardation (EUGR) caused by inadequate absorption of enteral nutrition. Currently, a large number of studies have shown that extrauterine growth retardation has definite influences on the subsequent cognition and mental development of preterm children [12].

Nutritional support in the neonatal period is one of the basic conditions for survival of preterm children. Preterm children born in advance for various reasons need parenteml nutrition if enteral nutrition cannot meet their needs for growth and development. Although parenteral nutrition supplement can meet the temporary nutritional needs of preterm children, long-term parenteral nutrition has brought a series of problems, such as difficulties in peripheral venous puncture, susceptibility to complication by infections after central venous catheterization, parenteral nutrition-associated cholestasis (PNAC) and other complications. The ultimate goal of nutritional support in preterm children is to not only reach the normal growth speed of normal fetuses of similar gestational age in utero, but also have similar composition and function to normal fetuses. The ideal growth rate of preterm/low-birth-weight children during the neonatal intensive care unit (NICU) stay, by referring to the growth rate of normal fetuses in utero [13], is required as follows: average daily weight gain of 15 g/kg, weekly body length growth of 1 cm and weekly head circumference growth of 0.5–1 cm. However, preterm children after birth are faced with respiratory, digestive and other problems. For preterm children with various immaturely developed systems, how to solve the conflict between the high nutritional demands and low gastrointestinal tolerability of preterm children is a very realistic and important issue. Therefore, we need an appropriate early enteral nutrition management strategy for preterm children, although there is already a lot of consensus [14, 15], such as breastfeeding, initiation of microfeeding as early as possible, achieving full enteral nutrition as soon as possible, and shortening the time of parenteral nutrition in order to reduce complications. It is preferred to feed preterm children with their mothers’ breast milk [14], because such feeding can reduce the incidence of feeding intolerance, neonatal necrotizing enterocolitis (NEC), nosocomial infections, and growth and developmental retardation in preterm children [16]. In the event of inadequate supply of breast milk due to various reasons or inability of breastfeeding due to maternal diseases, breast milk provided by qualified breast milk donors becomes the second-best nutritional source for preterm children [17] During October 2010 to December 212, a multicenter double-blind controlled study conducted in Canada [18] found that the breast milk of donors was equally safe and effective as the breast milk of preterm children’s mothers, and after long-term follow-up and study, found that donor breastfeeding was favorable for the development of the nervous system in preterm children. In Taiwan, China [19], the breast milk in the breast milk bank is mainly used for feeding preterm children, infants with feeding intolerance and other infants suffering from metabolic disorders. Currently, many developed countries have established large breast milk banks, and breast milk can be shared even via the Internet [20]. However, the breast milk in breast milk banks is mostly for full-term children, whose nutritional ingredients cannot meet the needs of growth and development of preterm children. Breast milk banks have not been set up in most areas of China, and no unified standard donor screening, breast milk collection, disinfection, preservation and feeding practices have been established. The rural population accounts for the majority in China. Rural areas and small cities lack the capacity to treat preterm children, so preterm children are often sent to NICU in large cities for hospitalization. Their homes are often far away from the hospital, and China’s NICUs are mostly accompany-free wards, so family members cannot participate in feeding and care. Besides, many mothers cannot breastfeed because of their illness, and thus, preterm children hospitalized in NICUs in China are mostly fed with formula.

In China, preterm children with a gestational age of less than 34 weeks are mostly fed with preterm children’s formula, and such milk powder provides sufficient energy, protein, vitamins and minerals and features a high growth rate in preterm children. However, excessive weight gain in early infancy will increase the risk of metabolic diseases and cardiovascular diseases in adulthood [21, 22]. The probabilities of feeding intolerance and neonatal necrotizing enterocolitis and overall mortality in preterm children fed with preterm children’s formula are significantly higher than in children fed with breast milk or donated breast milk [23–25]. Therefore, choosing a milk powder more suitable for the intestinal function of preterm children with a gestational age of less than 34 weeks is very important.

Hydrolyzed formula is to hydrolyze protein in milk powder to polypeptides, peptides and free amino acids with bio-lyase, heating, ultrafiltration and other special technologies. The American Academy of Pediatrics (AAP) refers to formula whose peptide molecular weight is less than 3000 Da as extensively hydrolyzed formula (EHF) [26]. According to the protein ingredient therein, the formula can be divided into hydrolyzed protein formula, hydrolyzed soy protein formula and hydrolyzed rice protein formula; according to the milk protein ingredient therein, the formula can be divided into hydrolyzed whey protein, hydrolyzed casein, and hydrolyzed whey protein/casein. The low-molecular-weight peptides and small amounts of free amino acids in extensively hydrolyzed milk protein formula have greatly reduced the conformation and sequence of allergen idiotope, and thus the antigenicity is greatly lowered. The milk powder is mainly used for the treatment of cow’s milk protein allergy (CMPA) [27]. Researchers have fed preterm children in their early stage of life with EHF to prevent the occurrence of CMPA [28, 29], but there is no conclusion on whether it is effective.

EHF has cracked milk protein to low-molecular-weight peptides and partial amino acids, favorable for the gastrointestinal tolerability of preterm children. Frati et al. [30] and Staelens et al. [31] found by adopting different methods that the gastric emptying of EHF was significantly faster than the ordinary formula group; Mihatsch et al. [32] used carmine as a fecal marker and found that in preterm children, the gastrointestinal transit time of extensively hydrolyzed formula was 9.8 hours, while that of standard ordinary formula was 19 hours. The main mechanisms that EHF is favorable for the gastrointestinal tolerability of preterm children are summarized as follows: (1) it promotes gastric emptying [30, 31, 33, 34]; (2) it accelerates gastrointestinal transit [32]; (3) it reduces the activity of opioid receptor agonist-milk protein [35]; and (4) it induces motilin (MOT) and gastrin (GAS) secretion [36], thereby reducing the incidence of gastroesophageal reflux (GER) [30, 34, 37], feeding intolerance, etc., in preterm children [38] so as to achieve full enteral nutrition as soon as possible and reduce the time of parenteral intravenous nutrition and a series of complications caused by parenteral nutrition.

Each 100 ml of the EHF used in this study can provide 66 kcal, whose protein-to-energy ratio (P/E) is 2.42 g/100 kcal, lower than the P/E recommended by the European Society for Paediatric Gastroenterology, Hepatology, and Nutrition (ESPGHAN) [39]. Can extensively hydrolyzed milk protein formula meet the high nutritional needs of preterm children? Current study findings have differing conclusions [40–44]. Some studies conclude, by detecting the concentrations of amino acids in serum and urine and intestinal absorption of nitrogen after preterm children are fed with different milk powders, that the protein content in hydrolyzed milk protein formula should be higher than ordinary preterm children’s formula in order to meet the needs of preterm children for growth and development [40–42]. However, these studies all have too small sample size (15 cases, 21 cases, 16 cases), the period of literature [40] study is too short (the conclusion is drawn from determination of plasma amino acid concentrations only after cross feeding with the two kinds of milk powder for 5 days), and in the above studies, the preterm children began to be fed with hydrolyzed formula after achieving full enteral feeding (EF) or 1 week after birth instead of feeding with hydrolyzed formula immediately after birth. Szajewska et al. [43] conducted a randomized controlled study of up to 12 weeks. The authors randomized low-birth-weight children for feeding with different milk powders, then tracked and monitored the growth and development (weight, body length and head circumference growths) and all plasma parameter indicators (urea, albumin (ALB), prealbumin, transferrin, and concentration of each amino acid) of these infants at the beginning of feeding, 4 weeks, 8 weeks and 12 weeks after feeding, and found that the nutrition of extensively hydrolyzed whey protein formula was equivalent to that of the standard preterm children’s formula. In China [44], a multicenter (eight domestic first-class grade 3 hospitals) controlled clinical study from February 2012 to December 2013 found that extensively hydrolyzed milk protein formula could promote the gastrointestinal motility of preterm children and accelerate their metabolism and excretion of bilirubin, without increasing the occurrence of EUGR at discharge. Besides, there is no study at home and abroad on whether feeding preterm children with relatively low-energy low-protein extensively hydrolyzed milk protein formula in the early stage of life will affect their subsequent growth and development. Although studies [28, 29] respectively followed up preterm children and extremely low-birth-weight children after feeding with EHF for the early stage of life for 12 months and 5–7 years, these two studies were conducted only to observe whether EHF could prevent the subsequent occurrence of CMPA in preterm children, without tracking and observing the growth and development of those preterm children.

The protein in the extensively hydrolyzed milk protein formula used in this study is 1 % whey protein, without containing casein. A study has found that the higher the casein content, the slower the gastric emptying [45]. Each 10 ml of the EHF we use provides 66 kcal with osmolality of 250 mOsmol/L, lower than traditional preterm children’s formula. Another study has also found that the higher the calories provided by each 100 ml of formula, the slower the gastric emptying [46]. Therefore, in this study, the gastric emptying time and gastrointestinal transit time in preterm children fed with extensively hydrolyzed milk protein formula will be accelerated, thereby reducing the incidence of feeding intolerance in the preterm children, achieving full enteral feeding as soon as possible, shortening the time of parenteral nutrition, and reducing the occurrence of complications associated with parenteral nutrition. This formula has cracked milk protein to low-molecular-weight peptides and partial amino acids, it reduces the intestinal load of protein digestion in preterm children, and is thus favorable for the gastrointestinal capacity of preterm children for protein digestion and absorption. Moreover, medium-chain triglycerides fat (MCT) in the formula accounts for 40 % of total fat, which is favorable for preterm children’s fat absorption and utilization. The energy and protein provided by each 100 ml of the milk powder are lower than traditional preterm children’s formula, but it may not affect the early nutrition of preterm children due to the abovementioned features. Can feeding preterm children with a gestational age of less than 34 weeks during the NICU stay with extensively hydrolyzed milk protein formula meet the preterm children’s needs for early normal growth and development during hospital stay and after discharge? In addition to observation of the preterm children’s growth and development during the NICU stay, our study will also track and follow up these preterm children for at least 3 months after they are discharged from the NICU.

## Methods/Design

A prospective, randomized, single-center, single-blind and controlled clinical trial will be conducted in preterm children with a gestational age of less than 34 weeks meeting the inclusion criteria who cannot be breastfed. The implementer of the study will be the NICU of Zhongda Hospital Southeast University. The majority of the center’s preterm children are transferred from other hospitals in Nanjing or nearby towns, wherein most preterm children who cannot be breastfed because their mothers suffer from infectious diseases or other severe diseases requiring long-term medication are also transferred to our hospital. Most preterm children hospitalized in our hospital’s NICU can only be fed with formula. Every year, about 2,000 newborns are hospitalized in our hospital’s NICU, and preterm children with a gestational age of less than 34 weeks account for 10–15 % of the inpatients, of whom about two thirds can only be fed with formula.

### Objectives

#### Primary objective

To determine whether feeding preterm children with a gestational age of less than 34 weeks during the NICU stay with extensively hydrolyzed milk protein formula can reduce the rate of food intolerance or not.

#### Secondary objective

To test if the feeding preterm children with a gestational age of less than 34 weeks during the NICU stay with extensively hydrolyzed milk protein formula can accelerate the time to achieve full enteral feeding, shorten the time of parenteral nutrition and NICU stay, benefit spontaneous fecal discharge, reduce the occurrence of other complications associated with parenteral nutrition, and meet the nutritional needs of preterm children during the NICU stay, without affecting the early growth and development of these preterm children during hospitalization and after discharge.

### Study population

#### Recruitment of participants

Preterm children hospitalized in the NICU of Zhongda Hospital Southeast University in line with the inclusion criteria are recruited as participants (Fig. 1).

Inclusion criteria:

1. Gestational age <34 weeks
2. Children who can only be fed with formula due to their mothers’ sickness
3. Children who are transferred to our hospital’s NICU immediately after birth, and have not started any enteral feeding before and during transfer, including water, glucose, formula, drugs, etc.
4. Children whose cardiovascular, blood and nervous systems are stable when transferred to the NICU

Exclusion criteria:

1. Occurrence of vomiting, abdominal distension, gastrointestinal hemorrhage or necrotizing enterocolitis (NEC) before preparation for enteral feeding
2. Occurrence of cardiovascular, blood and nervous system instability (such as shock, disseminated intravascular coagulation (DIS), frequent convulsions, coma) before preparation for enteral feeding
3. Discovery of gastrointestinal malformations (esophageal atresia, esophageal fistula, biliary atresia, Hirschsprung’s disease, anal atresia, omphalocele, gastroschisis, congenital malrotation, etc.) before preparation for enteral feeding or after feeding
4. Children with congenital metabolic diseases and chromosomal disorders
5. Children with other serious congenital disorders (diaphragmatic hernia, cyanotic congenital heart disease, moderate to severe Pierre-Robin syndrome, etc.)
6. Renal failure due to various causes
7. Children whose head computed tomography (CT) or B-mode head ultrasound prompts serious intracranial hemorrhage or hydrocephalus
8. Use of drugs affecting gastric motility and gastric acid secretion (H_2_ receptor antagonists, proton pump inhibitors, gastrointestinal drugs)
9. Occurrence of stage III necrotizing enterocolitis during hospital stay requiring transfer to a Children’s Hospital for observation and surgical treatment
10. Replacement for formula during hospital stay
11. Death during hospital stay
12. Early discharge against the criteria for discharge from the NICU
13. Children who suffer from diseases requiring parenteral nutrition or serious illness requiring intensive care unit (ICU) hospitalization or requiring hospitalization and surgery

Criteria for discharge from the NICU or transfer to direct rooming-in (DRI):

1. Corrected gestational age ≥34 weeks, and weight ≥1500 g
2. No need for oxygen inhalation, stable vital signs, no apnea phenomenon
3. Able to suck formula on their own, and coordinated sucking-swallowing-breathing action
4. Having achieved full enteral feeding, daily milk volume ≥150 ml/kg, no need for any intravenous medication
5. Able to maintain normal body temperature at room temperature of 22–24 °C
6. Each system is free from life-threatening pathological conditions
7. Birth weight has been restored, and weight begins to grow steadily

#### Randomization and blinding

Randomization will be conducted by a researcher not involved in the recruitment and treatment of the participants. Concealed allocation will be performed using a set of random numbers placed in sealed opaque envelopes. The participants who meet the eligibility criteria will be divided into two groups by the physicians on duty via opening the envelopes. Reference to similar study literature is made for randomization [28, 31]. As extensively hydrolyzed milk protein formula and preterm children’s formula are greatly different in color and smell, physicians and nurses can easily distinguish these two formulae. Therefore, the study has chosen single-blinding, which means that only the participants and their guardians are unaware of the milk type.

#### Ethical aspects and informed consent

The study has passed the review of the Clinical Study Ethics Committee of Zhongda Hospital Southeast University, and the Ethics Committee’s approval number is 2014ZDSYLL115.0. Only the preterm children whose guardians give informed written consent will be included in this trial.

#### Study time

The study started in November 2014 and is expected to end in November 2017.

#### Interventions

The observation group is fed with extensively hydrolyzed milk protein (100 % whey protein) formula, while the control group is fed with preterm children’s formula until discharge from the NICU. After discharge, both groups are uniformly fed with ordinary formula (formula for 0 to 6-month-old infants).

### Study design and outcomes

#### Outcomes

The first endpoint variable is the rate of food intolerance in preterm children. The second endpoint variables include (1) time to achieve full enteral nutrition; (2) time of parenteral nutrition; (3) time of NICU stay; (4) meconium drainage time; (5) daily spontaneous fecal discharge conditions; (6) growth and development conditions during the NICU stay and within 3 months after discharge, including daily weight growth rate, weekly body length growth rate and weekly head circumference growth rate; (7) different plasma parameter indicators before discharge from the NICU (total protein (TP), albumin, calcium (Ca), phosphorus (P), and alkaline phosphatase (ALP)). Other endpoint variables include the incidences of neonatal necrotizing enterocolitis, cholestasis, parenteral nutrition-associated cholestasis and congenital hypothyroidism (CH).

#### Study design

The study includes two stages. In stage I, the study observes the incidence of feeding intolerance, parenteral nutrition conditions, spontaneous fecal discharge conditions, growth and development conditions and the incidence of other preterm children’s complications in the preterm children in the two groups during the NICU stay. In this stage, different feeding methods and parenteral nutrition are regulated according to the gestational age, birth weight and days after birth of the preterm children, and unified diagnostic criteria and treatment methods for different complications are developed. In stage II, the study follows up the growth and development conditions of the preterm children in the two groups on a regular basis after discharge from the NICU.

#### Introduction to the formulae

The two kinds of formula for feeding during the NICU stay and the formula for feeding after discharge from the NICU are all Dumex, a brand of French Danone-Nutricia for early life nutrition. In this study, the gestational age of all preterm children when discharged from the NICU is all ≥34 weeks, and thus formula suitable for 0 to 6-month-old infants is uniformly chosen for them after discharge. The ingredients of such formulae are shown in Table 1.

**Table 1:** Table of ingredients of the three formulae

Ingredients	Preterm children’s formula (/100 ml)	Extensively hydrolyzed formula (/100 ml)	Formula for 0 to 6-month-old infants (/100 ml)
Energy (kcal/KJ)	80/336	66/278	67/282
Osmolality (mOsmol/L)	360	250	-
Fat (g)	3.8	3.5	3.5
α-linolenic acid (mg)	44	58	42
Linoleic acid (g)	0.6	0.41	0.42
Oleic acid (g)	1.3	1.2	-
Medium-chain triglyceride content (%)	30 %	40 %	-
Protein (g)	2.6 (60 % whey protein and 40 % casein)	1.6 (100 % whey protein)	1.4
Carbohydrates	8.4	6.9	7.1
Lactose (g)	5.6	2.8	-
Prebiotics (galactooligosaccharides, polyfructose) (g)	0.8	0.8	0.8
Arachidonic acid (mg)	19	6.6	12
Docosahexaenoic acid (mg)	15	6.6	12
Taurine (mg)	5.5	5.3	5.4
L-carnitine (mg)	1.8	1	1.5
Nucleotides (mg)	3.4	3.2	3.0
Vitamin A (μg)	359	52	65
Vitamin D (μg)	3	1.3	0.95
Vitamin E (mg)	3.5	1	1.2
Vitamin K1 (μg)	6	4.7	5.4
Vitamin B1 (μg)	139	50	58
Vitamin B2 (μg)	199	99	103
Vitamin B6 (μg)	119	40	48
Vitamin B12 (μg)	0.24	0.18	0.31
Nicotinamide acid (μg)	2352	432	517
Folic acid (μg)	35	9	11
Pantothenic acid (μg)	876	327	354
Vitamin C (mg)	17	9	9.2
Biotin (μg)	3.5	2.2	2.3
Choline (mg)	17	10	16
Inositol (mg)	24	3.2	3.8
Sodium (mg)	70	20	20
Potassium (mg)	80	75	69
Copper (μg)	80	40	48
Magnesium (mg)	8	5.1	5.7
Iron (mg)	1.6	0.53	0.71
Zinc (mg)	1.1	0.5	0.5
Manganese (μg)	10	7.4	7.5
Calcium (mg)	100	47	52
Phosphorus (mg)	56	26	31
Iodine (μg)	25	12	11
Chlorine (mg)	85	41	48
Selenium (μg)	4.5	1.5	2.2

### Stage I: NICU stay

1. Feeding time
  Preterm children without obvious high-risk factors are fed within 24 hours after birth.
  The feeding of preterm children with birth weight <1000 g, obvious history of asphyxia at birth (Apgar score ≤5), severe infection (sepsis, purulent meningitis, etc.), severe pneumonia or neonatal respiratory distress syndrome (NRDS) requiring intubation and mechanical ventilation should be postponed to 24–72 h after birth according to the situation.
  During the period when full enteral nutrition is not achieved, partial parenteral nutrition is given according to the daily amount of fluid and energy required by the children; when the children experience feeding intolerance and necrotizing enterocolitis and need to be fasting, complete parenteral nutrition is given; after the conditions are stable, the children gradually switch to enteral nutrition and are fed until discharge or transfer into DRI.
  Children in the two groups are fed with ordinary formula after discharge or transfer to DRI.
2. Feeding methods
  For preterm children with a gestational age of <32 weeks, since their sucking-swallowing action is uncoordinated and respiratory function is not maturely developed, the children are given tube feeding (nasogastric tube or orogastric tube) using the intermittent infusion feeding method. Every time before feeding, the stomach contents are drawn to learn about gastric residual volume (GRV) and nature.
  Children suspected of suffering from gastroesophageal reflux or delayed gastric emptying are fed by adopting intermittent/continuous infusion feeding method with an infusion pump.
  Preterm children with a gestational age of 32–34 weeks are given direct bottle feeding.
  Children with weak sucking ability yet normal gastric emptying are given bottle feeding first, and then the remaining milk is given by tube feeding.
  Children with nasal continuous positive airway pressure (nCPAP) or intubation and mechanical ventilation are given tube feeding;
  During tube feeding or fasting period, the children are given non-nutritive sucking (NNS).
3. Feeding milk volume
  Initial feeding milk volume: according to birth weight, the children are fed from different initial milk volume once every two hours, as shown in Table 2.
  The milk volume begins to increase in the absence of feeding intolerance in 24-h observation, and the rates for milk increasing are shown in Table 2.

**Table 2:** Initial feeding milk volume and increasing rate in the two groups of preterm children

Birth weight (g)	Initial feeding milk volume (ml/kg)	Increasing rate (ml/kg/d)
<1000	1.0	12
1000–1500	1.0–2.0	12–24
1501–2000	2.0	24–36
>2000	2.0	36
4. Fluid amount control [14, 47]

Fluid amount includes the total amount of enteral nutrition, parenteral nutrition and all other intravenous and oral medications. If birth weight is <1500 g, the fluid amount in the first 24 hours after birth is 80–100 ml/(kg/d); if birth weight is ≥1500 g, the fluid amount in the first 24 hours after birth is 60–100 ml/(kg/d); then the amount is incremented at a rate of 15–20 ml/(kg/d) to reach 150 ml/(kg/d) by the end of the first week; daily fluid amount is increased or decreased by 10–20 ml/kg on the basis of the fluid amount that day according to different clinical conditions (light therapy, incubator, ventilator, heart and lung function, urine amount, systemic edema conditions, biochemical monitoring indicators, etc.), usually no more than 180 ml/(kg/d).

5. Calorie control [14, 47]

Calorie includes the total calories provided by enteral feeding and parenteral nutrition. The calorie on the first day is 40 kcal/kg and then incremented at a rate of 10 kcal/(kg/d). Parenteral nutrition is stopped when enteral nutrition reaches 80–100 kcal/(kg/d), and enteral nutrition 100–120 kcal/(kg/d) is the criterion to achieve full enteral nutrition.

6. Parenteral nutrition (PN) [14, 47]
  Vessel selection: peripheral vein or central vein, central vein includes peripherally inserted central catheter (PICC) and umbilical venous catheter (PVC).
  Method: all children adopt “all in one” parenteral nutrition. The nutrient solution used for daily parenteral nutrition is uniformly prepared inside on a clean bench at the hospital’s Intravenous Nutrition Preparation Center according to the doctor’s instructions that day, and attention should be paid to the sequence of mixing during preparation. After preparation the solution is evenly infused via the infusion pump within 24 hours. The total amount of parenteral nutrition fluid and compounding ratio of all nutrients are calculated according to the child’s total fluid amount required that day, total calorie, milk intake the day before and possible total milk intake that day.
  Children’s compound amino acid (6 %) 1.0–1.5 g/(kg/d) is added within 24 hours after birth, which is later incremented up to 3.5–4.0 g/(kg/d) at a rate of 0.5 g/(kg/d); medium and long-chain fat emulsion 0.5 g/(kg/d) is added within 24 hours after birth, which is later incremented up to 3.0 g/(kg/d) at a rate of 0.5 g/(kg/d).
  Carbohydrates ≤15 g/(kg/d), providing 40–50 % of the total energy; amino acids ≤3.5–4.0 g/(kg/d), providing 15–20 % of the total energy, fat emulsion ≤3.0 g/(kg/d), providing 40–50 % of the total energy.
  Sodium ions 2.0–3.0 mmol/L and potassium ions 1.0–2.0 mmol/L are supplemented daily, but within 3 days after birth, no potassium is supplemented in principle unless the child is suffering from hypokalemia.
  A variety of water-soluble and fat-soluble vitamins 0.5–1.0 ml/(kg/d) are supplemented.
  If the child has cholestasis, the amount of intravenous fat emulsion is reduced.
  The concentration and rate of intravenous nutritive sugar are adjusted according to the child’s blood glucose monitoring. In case of PVC or PICC, the sugar concentration is ≤15 %; when peripheral intravenous infusion is adopted, the sugar concentration is ≤12.5 %. The child’s blood glucose is controlled at 3.0–7.0 mmol/L.
7. Diagnostic criteria for and treatment of various complications:
  1. Diagnostic criteria for and clinical treatment of feeding intolerance [48, 49]

Children in line with any of the following six criteria can be diagnosed with feeding intolerance:

- Gastric residual liquid >50 % of the amount of previous feeding
- Vomiting ≥3 times/day, or the vomitus is bile-like
- Gastric residual liquid or vomitus is bile-like or coffee grounds-like
- Abdominal distension (abdominal girth is increased by ≥1.5 cm within 24 hours, with intestinal type), and abdominal distension caused by the use of nCPAP should be ruled out
- Required fasting >2 meals
- Blood in the stool or fecal occult blood positive, but it is required to rule out NEC based on the general conditions, blood indicators and abdominal X-ray examination of children

Clinical treatments:

- Amount of gastric residue: for children with tube feeding, draw the residual milk in the stomach every time before feeding.
  - If birth weight is <1500 g, and gastric residue is <2 ml or if birth weight is ≥1500 g and gastric residue is <3 ml or gastric residue is <30 % of the amount of previous feeding, continue feeding and closely observe.
  - If gastric residue is 30–50 % of the amount of previous feed, reduce the amount of feeding.
  - If gastric residue is >50 % of the amount of previous feeding, fast for one meal, and continue feeding by the original feeding plan after the condition is improved.
  - If there is bile-like or coffee grounds-like liquid in the gastric residual liquid or vomitus, stop feeding.

- Abdominal distension: measure the abdominal girth at fixed parts every time before feeding. If the abdominal girth is increased by ≥1.5 cm, reduce the amount of feeding or fast for one meal, and continue feeding by the original feeding plan after the abdominal distension condition is improved.
- If daily vomiting occurs >3 times, do not increase the milk volume or reduce the amount of feeding.
- In the event of blood in the stool or fecal occult blood positive, stop feeding.

2. Diagnostic and treatment reference criteria for necrotizing enterocolitis (NEC) [50] are shown in Table 3.

**Table 3:** Modified Bell staging criteria for neonatal necrotising enterocolitis

Staging	Systemic symptoms	Gastrointestinal symptoms	Radiological signs	Treatment
IA Suspected NEC	Temperature instability, apnoea, bradycardia, lethargy	Gastric retention, mild abdominal distension, fecal occult blood positive	Normal or intestinal dilation, mild ileus	Absolute fasting, gastric decompression, antibiotic therapy for 3 days, waiting for pathogen culture results
IB Suspected NEC	Same as IA	Bright-red blood from rectum	Same as IA	Same as IA
IIA proven NEC (mildly ill)	Same as IA	Same as IA or IB, plus absent bowel sounds, and (or) abdominal tenderness,	Intestinal dilation, ileus, pneumatosis intestinalis	Same as IA, absolute fasting. If 24–48 h culture shows no abnormality, use antibiotics for 7– 10 days
IIB proven NEC (moderately ill)	Same as IIA, plus mild metabolic acidosis and mild thrombocytopenia	Same as IIA,plus absent bowel sounds, definite abdominal tenderness, and (or) abdominal cellulitis or right lower quadrant mass	Same as IIA, plus portal vein gas, and (or) ascites	Same as IIA, absolute fasting. Supplement blood volume, treat acidosis, and use antibiotics for 14 days
IIIA Advanced NEC (severely ill, bower intact)	Same as IIB, plus hypotension, bradycardia, severe apnea, mixed acidosis, DIC, neutropenia, anuria	Same as IIB, plus signs of generalized peritonitis, abdominal distension or marked tenderness, and redness and swelling of abdominal wall	Same as IIB, ascites	Transfer to the Surgical Department of Children’s Hospital for observation
IIIB Advanced NEC (severely ill, bowel perforated)	Same as IIIA, plus suddenly aggravation of conditions	Same as IIIA, plus sudden aggravation of abdominal distension	Same as IIB, pneumoperitoneum	Transfer to the Surgical Department of Children’s Hospital for surgery

*NEC* necrotising enterocolitis, *DIC* disseminated intravascular coagulation
3. Diagnostic criteria for and clinical treatment of cholestasis:
  Diagnostic criteria [51]: in line with any of the first two criteria:
  Total bilirubin (TB) <5 mg/dl and direct bilirubin (DB) >1.0 mg/dl
  TB >5 mg/dl and DB >20 % of TB
  Elevated total bile acid (TBA) levels alone cannot serve as a diagnostic criterion for cholestasis
4. Diagnosis and clinical treatment of parenteral nutrition-associated cholestasis (PNAC):

Diagnostic criteria for PNAC [52]:

- Continuous parenteral nutrition ≥14 days
- Serum DB >34umol/L (2 mg/d1)
- Clinical manifestations of yellowish discoloration of skin, hepatosplenomegaly, elevated transaminase levels and (or) lighter stool color
- Exclusion of other diseases causing cholestasis (various biliary malformations causing biliary obstruction, hereditary metabolic diseases and hepatitis caused by bacterial and viral infections)

Clinical treatment of PNAC:

- Protect the liver and gallbladder: ursodesoxycholic acid 15 mg/(kg/d), three times daily, by mouth
- Do not lightly fast; increase enteral nutrition, reduce the proportion of parenteral nutrition, and medium and long-chain fat emulsion ≤1 g/(kg/d)
- Additionally supplement vitamin E 10 mg/(kg/d), twice daily, by mouth

5. Diagnostic criteria for and clinical treatment of congenital hypothyroidism [53].

Diagnostic criteria: 1 week after birth, draw blood to test serum thyroid-stimulating hormone (TSH), total triiodothyronine (TT_3_), total thyroxine (TT_4_), free triiodothyronine (FT_3_) and free thyroxine (FT_4_). If blood test shows no abnormality but there are clinically suspicious symptoms of hypothyroidism, retest the thyroid function. Suspicious symptoms of preterm children’s hypothyroidism include persistent jaundice, lethargy, less crying, low and weak crying, slow heart rate, low and blunt heart sounds, poor digestion (weak sucking, feeding intolerance), recurrent hypoglycemia, etc.

- If blood TSH is >10 mU/L and FT_4_ is decreased, the child will be diagnosed with congenital hypothyroidism.
- If blood TSH is >10 mU/L and FT_4_ is normal, the child will be diagnosed with high TSH hyperlipidemia.
- If blood TSH is normal or decreased and FT_4_ is decreased, the child will be diagnosed with secondary or central hypothyroidism.

Clinical treatment:

- In line with criterion (1), levothyroxine (Euthyrox) from a starting therapeutic dose of 10–15ug/(kg/d), daily, by mouth.
- In line with criterion (2), retest 1 week later. If TSH is still >10 mU/L, give Euthyrox 4–8ug/kg/d, daily, by mouth.
- In line with criterion (3), retest 1 week later. If FT_4_ is still decreased, give Euthyrox 4– 8ug/kg/d, daily, by mouth.
- If TSH always maintains at 6–10 mU/L, without treatment, regularly follow up thyroid function.
- If FT_4_ and TSH are normal, children with decreased TT_3_ or TT_4_ are given no treatment.
- For children with abnormal test results, draw blood for retest in 1–2 weeks. Adjust the therapeutic dose based on the concentrations of blood TSH and FT_4_ and the children’s weight.

6. Diagnostic criteria for and clinical treatment of hypocalcemia [54]

Diagnostic criteria:

Total serum calcium is less than 1.75 mmol/L (7.0 mg/dl) or free calcium is less than 0.9mmom/L (3.5 mg/dl).

Clinical treatment:

- For children with clinical seizures or heart rhythm disorders, which cannot be explained by other diseases, give them slow intravenous injection of 10 % calcium gluconate 2 ml/kg, and use 5 % glucose injection after dilution by onefold. Then according to the retest result, decide whether to continue intravenous injection or switch to oral medication.
- For children with stable conditions, give them tube feeding or oral 10 % calcium gluconate.

### Stage II: follow-up period after discharge from the NICU

1. Develop a follow-up observation table for preterm children <34 weeks, and require the children’s health care medical staff to record the data of follow-up every time in detail.
2. Require the preterm children to revisit the hospital’s Child Health Clinic for referral 2 weeks, 4 weeks and 3 months after discharge, and follow up once every 2–4 weeks according to the preterm children’s conditions 4 weeks later.
3. Call the children’s parents 1–2 day before every referral and require them to bring the children to our hospital for referral on time, and ask them to bring all medical data.
4. Contents of referral at the Child Health Clinic include measurement of physical growth and development, assessment of neuropsychological development, nutritional assessment and feeding guidance. According to different conditions of the preterm children during hospital stay, choose whether to conduct blood, liver function, thyroid function, head B-mode ultrasound or cranial magnetic resonance imaging (MRI), retinopathy (ROP) screening, hearing screening and other tests.
5. Explain the current follow-up results in detail to the parents, and appoint the time and contents of next follow-up.
6. Call the parents again on the night or the next day of follow-up every time to inquire about follow-up conditions in detail, and again record all follow-up results.
7. Follow-up period: at least 3 months.

### Data collection and management

Including the collection of clinical data of preterm children during the NICU stay and follow-up data after discharge from the NICU.

### Collection of clinical data during the NICU stay

1. Collection of general data of the objects of study:
  - Sex, gestational age, birth weight, birth head circumference, birth body length, mode of delivery, 5-minute Apgar score, etc., of preterm children
  - Mother’s pregnancy complications and comorbidities: premature rupture of membrane >24 hours, placental abruption, chorioamnionitis, pregnancy-induced hypertension syndrome, preeclampsia, gestational diabetes, hyperthyroidism, hypothyroidism, systemic lupus erythematosus, kidney disease, cholestasis, acute liver failure, syphilis infection, etc.
  - Special treatment: nasal continuous positive airway pressure (nCPAP), intubation and mechanical ventilation (MV), use of pulmonary surfactant (PS), thoracentesis, bone marrow biopsy, thoracic drainage, peritoneal dialysis, etc.
2. Collection of case data during the NICU stay:

Incidence and outcome of feeding intolerance in preterm children, neonatal necrotizing enterocolitis, cholestasis, parenteral nutrition-associated cholestasis and congenital hypothyroidism

3. Collection of enteral and parenteral nutrition and defecation conditions during the NICU stay:
  - Milk opening time (h), total amount of daily milk (ml) and calories (kcal), total amount of daily parenteral nutrition (ml) and calories (kcal), total volume of daily fluids (ml) and total calories (kcal)
  - The number of daily bowel movements, times of daily enema, trait and color of stool every time, meconium drainage time (h)
  - Gastric residual volume (ml) and nature, times and nature of vomiting, and abdominal distension
  - Time to achieve full enteral nutrition (d), and total time of parenteral nutrition
4. Collection of physical growth and development indicators during the NICU stay:
  - Weight: weight will be measured at admission, once every day during hospital stay and before discharge with a baby electronic scale, and the data will be accurate to 10 g.
  - Body length: body length will be measured at admission, once every week during hospital stay and before discharge, and the data will be accurate to 0.1 cm.
  - Head circumference: head circumference will be measured at admission, once every week during hospital stay and before discharge, and the data will be accurate to 0.1 cm.
5. Collection of laboratory indicators during the NICU stay:
  - Daily monitoring of peripheral blood glucose and transcutaneous bilirubin
  - Weekly detection of blood, liver and kidney function (total protein, albumin, total bilirubin, direct bilirubin, alkaline phosphatase, bile acids, alanine aminotransferase, aspartate aminotransferase, γ-glutamyl transferase, urea nitrogen, creatinine) and electrolytes (mainly calcium and phosphorus)
  - Test of serum thyroid function 1–2 weeks after admission, including TSH, TT_3_, TT_4_, FT_3_ and FT_4_

### Collection of follow-up data after discharge from the NICU

1. Weight: weight will be measured once 2 weeks, 4 weeks and 3 months after discharge, respectively, and the data will be accurate to 10 g.
2. Body length: body length will be measured once 2 weeks, 4 weeks and 3 months after discharge, respectively, and the data will be accurate to 0.1 cm.
3. Head circumference: head circumference will be measured once 2 weeks, 4 weeks and 3 months after discharge, respectively, and the data will be accurate to 0.1 cm.

### Sample size justification

We calculate the sample size based on the chi-square test comparison of the food intolerance rate during NICU between groups. Our previous pilot study shows that the rate of food intolerance during NICU for control patients is about 85 %. We expect that the intervention group will have a reduced intolerance rate to be 75 %. With these assumptions, we will need 148 infants in each group to finish the NICU part of the study to reach 80 % power and 5 % Type I error rate. Assuming that the dropout rate to be 20 %, we will need to enroll 185 infants in each group.

### Statistical analysis

#### Preliminary analysis

Patients’ characteristics will be summarized using mean ± SD for continuous variables and frequency (percentage) for categorical variables by groups. Continuous variables will be transformed if there is a normal assumption violation.

#### Primary outcome

The rates of food intolerance during NICU will be summarized and compared using the chi-square test. A *p* value <0.05 indicates a significant difference in rates between the two groups. We will also fit the logistic regression model with food intolerance status as the outcome variable, and group as the main predictor adjusting for covariates. In the case that the number of intolerance events is large enough, we will consider using a Poisson model to model the number of intolerances.

#### Secondary outcome

The time to reach enteral nutrition (parenteral nutrition), length of hospital stay, meconium drainage time during the NICU stay will be summarized and compared using Kaplan-Meier estimators and logrank tests. Cox models will also be fitted for time-to-event data adjusting for baseline covariates. The number of times of self-defecating per day will be compared using the Poisson model adjusting for baseline covariates. The rates of comorbidity will be compared using Poisson models. The plasma total protein, albumin level, calcium, alkaline phosphatase levels before discharge from the NICU will be compared using *t* tests. Growth rates measure by weight, body length, and head circumference during the NICU and within 3 months of discharge will be analyzed using repeated measurement analysis of covariance models. All analysis will be conducted using SAS 9.4 (SAS Institute, Cary, NC, USA).

## Discussion

Extensively hydrolyzed milk protein formula can accelerate gastric emptying and gastrointestinal transit time, and promote gastrointestinal hormone secretion, and these mechanisms are conducive for the gastrointestinal tolerability of preterm children, thereby lowering the incidence of gastroesophageal reflux and feeding intolerance in preterm children, achieving full enteral nutrition as soon as possible and reducing the time of parenteral nutrition. In China, more and more medical organizations have started using extensively hydrolyzed milk protein formula to feed preterm children, but so far there is no clear conclusion on whether low-energy low-protein extensively hydrolyzed formula can meet the high nutritional needs of preterm children in the feeding period, and there is a lack of relevant studies and conclusions on whether feeding with extensively hydrolyzed milk protein formula in the early stage of life will affect the subsequent growth and development of preterm children. As the conflict between the high nutritional needs and low gastrointestinal tolerability of preterm children with a gestational age of less than 34 weeks is an unavoidable issue, we have designed this study protocol, aiming to increase a new and truly safe and effective enteral nutritional prescription. Our study observes the effect of extensively hydrolyzed milk formula on the feeding intolerance, enteral and parenteral nutrition, spontaneous fecal discharge and other complications in preterm children with a gestational age of less than 34 weeks during the NICU stay; it also is the first controlled clinical study with long-term observation of the growth and development of preterm children fed with such formula in the early stage of life. Our study time includes NICU stay and 3-month follow-up after discharge of preterm children. The results of this study will confirm that feeding preterm children with such formula during the NICU stay is safe and effective, and does not affect the normal growth and development of preterm children in the early stage of their lives.

## Trial status

We started the enrollment in November 2014 and the trial is expected to end in November 2017.

## Abbreviations

AAP: American Academy of Pediatrics
ALB: serum albumin
ALP: alkaline phosphatase
Ca: calcium
CH: congenital hypothyroidism
CMPA: cow’s milk protein allergy
CT: computed tomography
DB: direct bilirubin
DRI: direct rooming-in
EF: enteral feeding
EHF: extensively hydrolyzed formula
ESPGHAN: European Society for Pediatric Gastroenterology, Hepatology, and Nutrition
EUGR: extrauterine growth restriction
FI: feeding intolerance
FT_3_: free triiodothyronine
FT_4_: free thyroxine
GAS: gastrin
GER: gastroesophageal reflux
GRV: gastric residual volume
LCT: long-chain fatty acids
MCF: medium-chain triglycerides fat
MMC: migrating motor complex
MOT: motilin
MRI: magnetic resonance imaging
MV: mechanical ventilation
nCPAP: nasal continuous positive airway pressure
NEC: necrotising enterocolitis
NICU: Neonatal Intensive Care Unit
NNS: non-nutritive sucking
NRDS: newborn respiratory distress syndrome
P: phosphorus
P/E: protein-to-energy ratio
PICC: peripherally inserted central catheter
PN: parenteral nutrition
PNAC: parenteral nutrition-associated cholestasis
PS: pulmonary surfactant
PVC: percutaneous umbilical vein catheter
ROP: retinopathy
TB: total bilirubin
TBA: total bile acid
TP: total protein
TSH: thyroid-stimulating hormone
TT_3_: total triiodothyronine
TT_4_: total thyroxine
WHO: World Health Organization
